# Feeding Behaviour on Host Plants May Influence Potential Exposure to *Bt* Maize Pollen of *Aglais Urticae* Larvae (Lepidoptera, Nymphalidae)

**DOI:** 10.3390/insects6030760

**Published:** 2015-08-31

**Authors:** Andreas Lang, Mathias Otto

**Affiliations:** 1 Environmental Geosciences, University of Basel, Bernoullistrasse 30, Basel CH-4056, Switzerland; 2 Büro Lang, Hörnlehof, Gresgen 108, Zell im Wiesental D-79669, Germany; 3 Federal Agency for Nature Conservation, Konstantinstraße 110, Bonn D-53179, Germany; E-Mail: ottom@bfn.de

**Keywords:** genetically modified plants, transgenic crop, pollen drift, risk assessment, non-target butterfly

## Abstract

Non-target butterfly larvae may be harmed by feeding on host plants dusted with *Bt* maize pollen. Feeding patterns of larvae and their utilization of host plants can affect the adverse *Bt* impact because the maize pollen is distributed unequally on the plant. In a field study, we investigated the feeding of larvae of the Small Tortoiseshell, *Aglais urticae*, on nettles, *Urtica dioica*. Young larvae used smaller host plants than older larvae. In general, the position of the larvae was in the top part of the host plant, but older larvae showed a broader vertical distribution on the nettles. Leaf blades and leaf tips were the plant parts most often consumed. Leaf veins were consumed but midribs were fed on to a lesser extent than other plant veins, particularly by young larvae. The feeding behavior of the larvae may increase possible exposure to *Bt* maize pollen because pollen densities are expected to be higher on the top parts and along leaf veins of nettles.

## 1. Introduction

In the European Union, genetically modified (GM) crops are regulated [1]. Thus, the release of GM crops in the environment can only be warranted after an environmental risk assessment. Insect resistance is one of the most frequent traits in transgenic crops [2]. Presently, insect resistance is mainly conferred by the insertion of genes from the soil bacterium *Bacillus thuringiensis* (*Bt*). Such *Bt* plants express insecticidal Cry proteins which, in a number of cases, are directed against pest moth species (Lepidoptera) [3]. In *Bt* maize, Cry proteins are expressed in most plant tissues including pollen, e.g., [4,5]. As maize pollen is dispersed by wind, the pollen can be deposited near maize fields, on the host plants of larvae of non-target butterflies e.g., [6,7,8,9]. Non-target butterfly larvae may feed plant material dusted with *Bt* maize pollen, thus being harmed sub-lethally or lethally, e.g., [10,11,12,13].

Concerns have been raised that the cropping of *Bt* maize may harm butterfly populations [10,14], and effects of *Bt* maize on non-target Lepidoptera are to be evaluated during the risk assessment and may trigger risk management measures, e.g., [15,16,17,18]. The risk is considered the probability that some adverse effect occurs from an environmental hazard, and, classically, the risk is comprised of (i) the probability that the environment is exposed to the hazard (exposure assessment); and (ii) the probability that the adverse effect will occur, given such exposure (effects assessment) e.g., [19]. The overall risk assessment procedure is then made up by four steps: hazard identification, exposure assessment, effects assessment, and risk characterization [20].

The adverse *Bt* maize impact realized in the field will depend on many factors such as the adoption rate of *Bt* maize by farmers, the Cry amount in the pollen of the respective *Bt* maize event, the susceptibility of the respective lepidopteran larvae or instar to Cry proteins, and the exposure of lepidopteran larvae to maize pollen, e.g., [14,15]. The exposure of the larvae is determined by multiple factors such as the distribution of their host plants relative to *Bt* maize fields, the pattern and distance to which maize pollen is dispersed in the landscape, the pollen density and pattern of pollen deposited on host plants, and the spatial distribution and feeding behavior of the larvae [8,21,22,23,24,25].

The specific feeding behavior of larvae can affect their exposure to *Bt* maize pollen because the maize pollen is not evenly distributed on host plants. Pollen will be deposited in different densities on different parts of the plants, e.g., accumulate along the midrib of leaves or differ between upper and lower leaves of a host plant [6,7,26,27]. Field studies estimating the potential exposure of butterfly larvae to *Bt* maize are rare [28,29]. In particular, data on larval activity and feeding patterns and their concurrent influence on *Bt* maize pollen exposure are lacking [25].

Nettles (*Urtica dioica* L.) commonly grow in farmland and can often be found along margins of maize fields [28,29,30,31]. Nettles serve as a host plant for various larvae of Lepidoptera species, including the Small Tortoiseshell, *Aglais urticae* (Linnaeus, 1758) [31], and have been used as a model for assessing *Bt* maize effects on non-target Lepidoptera [25,32]. The Small Tortoiseshell is a common butterfly across Europe, and the adults emerge from hibernation in spring and will reproduce shortly after, giving rise to one subsequent generation in North Europe, or two generations in warmer regions of Europe [33,34]. The larvae live and feed on nettles, and while the younger larvae aggregate in webs, the later instars will segregate [34]. Larval webs and older larvae are conspicuous and can be easily detected in the field. *A. urticae* larvae have been demonstrated to be susceptible to the Cry1Ab in *Bt* maize pollen [35]. The second larval generation will overlap with maize anthesis and thus be exposed to maize pollen [23]. Here, we studied larvae of the Small Tortoiseshell on nettles in the field in order to identify their behavior with regard to their spatial utilization of host plants and feeding patterns, and conclude on consequences for possible exposure to *Bt* maize pollen.

## 2. Experimental Section

### 2.1. Study Sites and Nettle Patches

Nine different patches of nettles (*Urtica dioica*) were studied, with seven patches in Baden-Württemberg, Germany, and two patches in Tirol, Austria. The two sites in Germany were located near Steinen (47°38'31.70'' N, 7°42'40.17'' E) and Todtnau (47°50'27.53'' N, 7°58'49.47'' E). The two sites in Austria were near St. Sigmund (47°12'2.47'' N, 11°6'2.49'' E) and Praxmar (47°9'9.58'' N, 11°8'2.76'' E). Field sampling took place in 2011, 2012, and 2013, between June 12 and August 1. Thus, representativeness of the study is obtained by covering nine nettle batches from four sites of two different countries and different years. All sites were situated in or at the margin of grassland grazed by cattle and were only sampled once. A summary of the study sites and nettle patches is provided in Table 1.

**Table 1:** Descriptives of the sampled sites in Germany (G) and Austria (A) (mean ± SE).

Sites	Sample Years	No. of Nettle Patches	Size of Patches (m^2^)	Nettle Density (Plants/m^2^)
Steinen (G)	2011, 2013	6	22.67 ± 4.58	118.17 ± 11.32
Todtnau (G)	2013	1	20	45
St. Sigmund (A)	2012	1	8	50
Praxmar (A)	2012	1	3	120

The average area of all nettle patches was 18.56 m^2^ ± 10.46 m^2^ (range: 1 m^2^–100 m^2^), with 102.67 ± 27.19 nettle plants growing per m^2^ (range: 42–250 nettles per m^2^) (arithmetic mean ± SE). Within each patch, 10 nettle plants were selected systematically along a linear gradient. About 60% of all checked nettles (*n* = 90) were flowering. The following data were recorded for each plant: the height of the nettle plant (cm), the number of leaves (*n*), and the heights (cm) of the lowest and highest leaf. The selected nettle plants showed little or no feeding damage, and were not occupied by butterfly larvae. Undamaged plants were chosen to characterize representative nettles of the patch in order to enable a comparison with nettles used by the larvae, *i.e.*, to analyze if the larvae prefer specific nettles other than the typical ones.

### 2.2. Butterfly Larvae in the Host Plant Patches

Only larvae of the Small Tortoiseshell, *Aglais urticae*, were recorded and studied. For each larva, the following parameters were noted: height of the occupied host plant (cm), height of larval position (cm), larval instar (L1–L5), body length of larvae (cm), location of the larvae on host plant (stem, leaf base, leaf blade, leaf apex, plant tip). In case the larvae sat on the leaf, it was noted if on the up-side or the underside. The larval stage was assessed by body length, according to own experience with *A. urticae* cultures kept and raised in the laboratory. For the analysis, the larvae were separated into “young larvae” (L1–L2) and “older larvae” (L3–L5). The younger larvae were all aggregated in batches, while the older larvae all occurred segregated. Each young larva was counted as an independent data entry. It may be argued that the data of young larvae are not independent because the younger instars were grouped. Still, we chose to analyze younger instars separately because each single larva would be exposed to an adverse external factor such as *Bt* maize pollen (or any other factor), thus representing a unique case in a risk assessment. For control, we repeated the analysis with average values for each batch of young larvae: the patterns and results were not different as compared to the analysis of the single larvae (Mann-Whitney U test, *p* > 0.05).

### 2.3 Feeding Patterns of Butterfly Larvae

**Figure 1:**
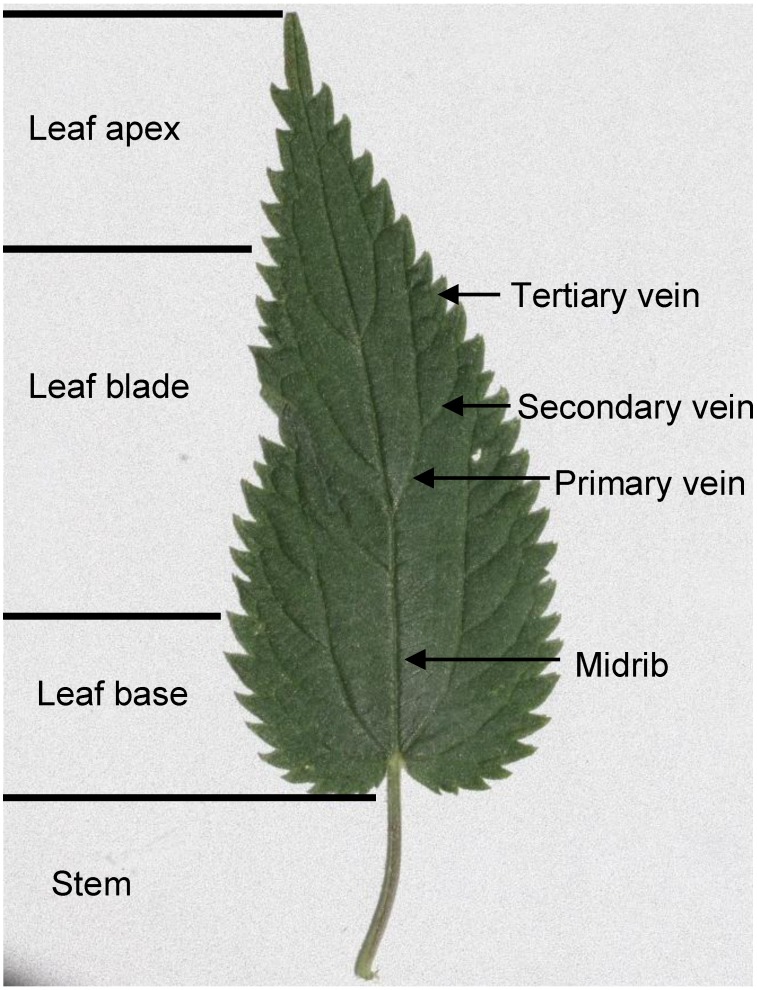
Classification of the surface area of a nettle leaf for the study of larval feeding patterns.

In each patch, parameters for nettle plants affected by feeding of *A. urticae* larvae were documented: height of the nettle plant (cm), proportion of nettle leaf material fed by larvae (%), location of larval feeding (stem, leaf, plant tip). For the leaves affected, the vertical position of the leaves (height in cm) and the surface area of consumed leaves (%) were documented. The percentage of nettle leaf material consumed by larvae was estimated by eye, using categories in 10% steps. In addition, it was recorded at what frequency (%) certain leaf areas and structures were affected by larval feeding. For this purpose, the leaf was divided into leaf stem, leaf base (the basic 25%), leaf blade (the 50% in the mid), and leaf apex (the top 25%), and the leaf structures “midrib”, “primary vein”, secondary vein”, and “tertiary vein” identified (Figure 1).

## 3. Results

### 3.1. Larvae in the Host Plant Patches

**Figure 2:**
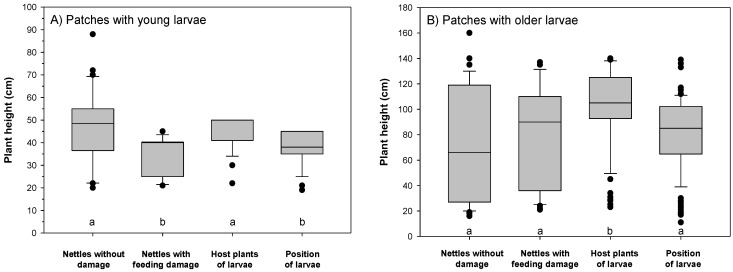
Height (cm) of nettle plants in the studied sites for patches with young larvae (**A**); and for patches with older larvae (**B**). “Nettles without damage” are plants without lepidopteran larvae (in patches with younger larvae, 30 nettles were recorded, and in patches with older larvae, 60 nettles). “Nettles with feeding damage” are plants with recorded feeding damage of lepidopteran larvae (in patches with younger larvae, 14 occupied nettle plants were recorded, and in patches with older larvae, 35 occupied nettles). “Host plants of larvae” refer to the specific nettles on which the Small Tortoiseshell larvae themselves were observed (several larvae can occur on the same nettle, but as each larva represents a single data entry, the sample size is 163 for young larvae and 118 for older larvae). “Position of larvae” corresponds to the height of each Small Tortoiseshell larvae on their respective host plant (*n* = 163 young larvae, and *n* = 118 older larvae). Boxes show the 25% and 75% quartiles, the horizontal line within the box is the median, while 10% and 90% percentiles are indicated by the whiskers, and outliers by dots. Boxplots with different letters below boxes differ significantly (*p* < 0.05, Dunn’s test).

For all following analyses, the data of the three sample years were pooled. Overall, 281 larvae of the Small Tortoiseshell were observed. In three nettle patches, 163 young larvae (L1–L2) of the Small Tortoiseshell were observed, and 118 older larvae (L3–L5) in six nettle patches. Younger larvae of *A. urticae* were found in patches with smaller nettle plants as compared to older larvae (Mann-Whitney U-test, *p* = 0.052, Figure 2). Both young and older larvae were mainly positioned at the upper part of their host plants (Figure 2), but significantly below the plant top (*p* < 0.05). Hence, the larger the nettle plant, the higher the vertical position of the larvae (r = 0.98, *p* < 0.001, *n* = 281, Spearman rank correlation). However, older larvae were distributed over a broader vertical range, and their relative positions were lower compared to the top of their respective host plants (Figure 2B, *p* < 0.05). The recorded vertical locations of feeding damage corresponded with the position of the larvae, indicating that the larvae did actually feed at the locations where they were observed. Younger larvae had a tendency to feed on smaller nettle plants, while older larvae tended to feed on taller nettles as compared to other undamaged nettles within the sites (Figure 2).

**Figure 3:**
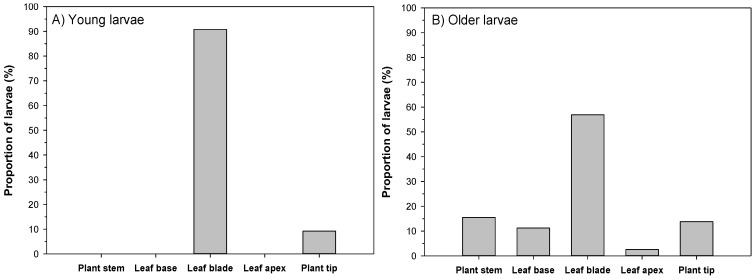
Locations of young larvae (**A**); and older larvae (**B**) of the Small Tortoiseshell (*A. urtica*) on nettle plants (N_young_ = 163, N_older_ = 116).

On the host plant, younger larvae sat predominantly on the leaf blade (91% of all cases), and only a small proportion of 9% of the larvae were found right at the tip of the nettle plants (Figure 3A). Older larvae preferred the leaf blades likewise (57% of the cases), but were also found on several other structures of the nettles, such as the stem (15%), leaf base (11%), leaf apex (3%), and plant tip (14%) (Figure 3B). Of the larvae located on the leaves, 66% of the young larvae were observed on the up-side of the leaf and 34% underneath, while the distribution for older larvae was 99% on the up-side and 1% on the underside of the leaf.

### 3.2. Feeding Patterns of Larvae

Overall, the feeding damage induced by *A. urticae* larvae were recorded on 49 nettle plants: 14 plants in patches with young larvae, and 35 plants in patches with older larvae. The heights of these plants were 34.64 ± 2.22 cm (young larvae) and 82.83 ± 6.46 cm (older larvae). In all cases, the main leaves and stipule leaves were fed on by larvae, but never the stem. The host plant tips were fed on in 50% of nettle plants by young larvae, and in 57% of the cases by older larvae. Young larvae consumed 27.14% ± 4.50% of the total main leaf material of each plant, and 45.83% ± 9.77% of stipule leaves. Older larvae consumed 31.39% ± 5.25% of total main leaf material, and 25.03% ± 5.85% of stipule leaves (arithmetic means ± standard error in all cases).

Feeding pattern was studied in more detail for the main leaves (Table 2). The studied leaves were positioned at a height of 31.39 ± 0.90 cm for young larvae (range 7–44 cm, *n* = 66 leaves), and at a height of 60.80 ± 2.39 cm for older larvae (range 13–131 cm, *n* = 164 leaves). Young larvae fed off 65.45% ± 3.98% of the surface area per main leaf (range 10%–100%), and older larvae fed off 48.34% ± 2.68% of each main leaf area (range 5%–100%) (arithmetic means ± standard error in all cases). All leaf structures were affected by larval feeding, the leaf blade and the apex most often including the leaf veins of these parts (Table 2). The thicker midrib and primary veins were fed on the least, but more often so by older larvae as compared to young larvae.

**Table 2:** Frequency of nettle leaf structures affected by larval feeding of *A. urticae* (young larvae = 66 leaves; older larvae = 164 leaves).

Leaf Structure	Young Larvae	Older Larvae
Leaf base	86%	61%
Leaf blade	92%	87%
Leaf apex	91%	85%
Midrib	17%	26%
Primary vein	68%	82%
Secondary vein	98%	98%
Tertiary vein	89%	87%

## 4. Discussion

The larvae of the Small Tortoiseshell sat predominantly on the top parts of their nettle host plants and on the up-sides of the leaves, which was even more pronounced for young larvae. This behavior will expose the larvae to the sun, speeding the developmental rate through higher temperatures [33,36]. Presumably, a faster development reduces larval mortality as the risky period of predation is shortened for this sensitive stage [37,38]. Young larvae occupied patches with smaller nettles in accordance to the adult females’ oviposition preference for freshly grown nettles [33]. Fresh nettles, e.g., nettles in spring or re-growing plants shortly after a mowing event, have a higher content of water, nitrogen, and protein, supporting a faster growth and greater weight of larvae feeding on such nettle plants [39,40]. The segregated older larvae showed a broader vertical range and also used lower parts of the nettles. In this study, the larvae of the Small Tortoiseshell started to segregate during the third instar, which is in contrast to other reports that *A. urticae* larvae do not separate before the fourth or fifth instar [34,41].

Only a portion of the dispersed maize pollen will be deposited on host plants, and the pollen will be unequally distributed on the plants [6,7,27,42,43]. On nettles, the upper parts of the plant will receive more maize pollen [44], and pollen often accumulates along leaf veins, especially on the midrib [6,7,26,43]. As the larvae were found and fed mainly on the tip of the nettles, this behavior will increase the exposure to *Bt* maize pollen. This may affect young larvae in particular because these are more sensitive to *Bt* [35] and tend to concentrate more at the plant top than older larvae do. The young larvae aggregate and feed in batches enclosed within a sort of web, which may affect the exposure of the larvae to pollen. However, in most of the observed cases, the complete nettle tips were consumed by the young larvae. Thus, the larvae would at least consume all pollen deposited on that part of the plant before they built their webs.

The leaf blade was most often fed on, which is in accordance with previous observations [29]. The larvae fed on all plant veins including midribs (one-fifth to one quarter of all cases). Feeding on midribs and primary veins was less frequent, presumably because these structures were too hard for the larval mouthparts, especially for the small first instars. Feeding on plant veins is relevant for the risk assessment of *Bt* maize because larvae feeding on these structures are subjected to higher *Bt* maize pollen densities as compared to the mean pollen densities per leaf blade [7,43]. Our results clearly show that feeding on leaf veins and midribs has to be taken into account in an exposure assessment for lepidopteran larvae. So far, feeding on leaf veins and midribs was considered to be negligible for the risk assessment [6,29], hence it was argued incorrectly that exposure estimates based on average leaf pollen densities would overestimate the exposure of larvae. It is worth noting that we just recorded the occurrence and frequency of vein and midrib feeding, but cannot conclude on the exact numbers of larvae that actually did so. The Cry proteins within the maize pollen will not be degraded by UV light quickly [45], thus *Aglais urticae* larvae will be exposed continuously to the toxin during their 2–3 weeks developmental period as long as the pollen is present on the host plant. How long maize pollen will remain deposited on host plants depends on weather conditions, as rain and wind can wash off the pollen [6,46].

Regional differences will exist as to the proportion of the butterfly larvae exposed to *Bt* maize pollen drift, and one important factor is the presence and spatial distribution of butterfly host plants in relation to *Bt* maize fields [28,29]. However, as nettles generally grow in margins of arable fields [30], any lepidopteran larvae feeding on nettles would potentially be at risk through *Bt* maize cultivation. Adult female *A. urticae* prefer fresh nettle plants, which often grow in field margins due to regular mowing, for oviposition [28,33]. As most of the maize pollen is deposited near the field edge, e.g., [42], the females’ oviposition preference for nettles in such margins would increase larval *Bt* maize pollen exposure. The shown feeding patterns of *A. urticae* larvae will also contribute to increased larval exposure to *Bt* maize pollen. As far as it is known, the larvae of the Peacock butterfly, *Inachis io* (Linnaeus, 1758), behave similarly to *A. urtica* larvae, with the possible exception that the Peacock seems to prefer nettles in more humid and shaded sites [33,37,38,39,47,48,49,50]. The behavior of the larvae of further lepidopteran species feeding on nettles is unknown, let alone of larvae utilizing host plants other than nettles.

## 5. Conclusions

Feeding patterns of butterfly larvae on their host plants can affect the exposure to *Bt* maize pollen. For example, larvae such as *Aglais urticae* feeding on the tip of their host plants would be more exposed to *Bt* maize pollen, given a pollen density gradient decreasing from tip to base of the respective plant. Predominantly feeding on the nettle leaf blade would presumably reduce the exposure of *Aglais urticae* larvae, while feeding on leaf veins would increase the exposure to *Bt* maize pollen as pollen are expected to aggregate in such structures.

Better in-depth knowledge of the specific feeding behavior of more lepidopteran species will provide a better understanding of the larval exposure by combining the larval feeding pattern with maize pollen densities on different parts of the host plants. Any improved information on larval exposure will support the final risk assessment.
